# Characterization of *Brucella abortus* mutant strain Δ22915, a potential vaccine candidate

**DOI:** 10.1186/s13567-017-0422-9

**Published:** 2017-04-04

**Authors:** Yanqing Bao, Mingxing Tian, Peng Li, Jiameng Liu, Chan Ding, Shengqing Yu

**Affiliations:** 1 Shanghai Veterinary Research Institute, Chinese Academy of Agricultural Sciences (CAAS), Shanghai, China; 2 Jiangsu Co-innovation Center for Prevention and Control of Important Animal Infectious Diseases and Zoonoses, Yangzhou, China

## Abstract

*Brucellosis*, caused by *Brucella* spp., is an important zoonosis worldwide. Vaccination is an effective strategy for protection against *Brucella* infection in livestock in developing countries and in wildlife in developed countries. However, current vaccine strains including S19 and RB51 are pathogenic to humans and pregnant animals, limiting their use. In this study, we constructed the *Brucella abortus* (*B. abortus*) S2308 mutant strain Δ22915, in which the putative lytic transglycosylase gene *BAB_RS22915* was deleted. The biological properties of mutant strain Δ22915 were characterized and protection of mice against virulent S2308 challenge was evaluated. The mutant strain Δ22915 showed reduced survival within RAW264.7 cells and survival in vivo in mice. In addition, the mutant strain Δ22915 failed to escape fusion with lysosomes within host cells, and caused no observable pathological damage. RNA-seq analysis indicated that four genes associated with amino acid/nucleotide transport and metabolism were significantly upregulated in mutant strain Δ22915. Furthermore, inoculation of ∆22915 at 10^5^ colony forming units induced effective host immune responses and long-term protection of BALB/c mice. Therefore, mutant strain ∆22915 could be used as a novel vaccine candidate in the future to protect animals against *B. abortus* infection.

**Electronic supplementary material:**

The online version of this article (doi:10.1186/s13567-017-0422-9) contains supplementary material, which is available to authorized users.

## Introduction

Brucellosis is a zoonotic disease epidemic in Asia, South and Central America, and sub-Saharan Africa [1]. It is caused by the genus *Brucella*, which infects millions of livestock and more than half a million people annually [2, 3]. Infection leads to reduction of animal productivity and debilitating disease in humans and causes economic losses and public health threats. Currently, vaccination of healthy animals is an effective strategy for protecting livestock from *Brucella* infection in developing countries and protecting wildlife in developed countries [4]. Vaccine strains such as S19, RB51 and Rev.1 have been extensively applied over the past decades with promising effects. These results stress the value of live attenuated vaccines. However, residue pathogenicity to humans and pregnant animals, and potential virulence reversion risks require the development of safer and better vaccines [5, 6].

Site-directed, unmarked deletion is an effective method for identifying virulence genes and constructing attenuated strains as *Brucella* vaccines. For example, acid shock protein 24 (*asp24*), ATP-binding/permease protein (*cydC*, a component of the cydDC operon), phosphoribosylamine-glycine ligase (*purD*), nitric oxide reductase activation protein (*norD*), high-affinity zinc uptake system (*zunA*), sigma factor (*rpoE1*, σ^E1^) and teichoic acid ABC transporter ATP-binding protein (*BAB_RS18515*) are involved with *Brucella* virulence [7–13]. Deletion of these genes reduces *Brucella* virulence, but they maintain excellent immunogenicity to activate the host immune response. These mutants provide protection against wild-type, virulent *Brucella* challenge in mouse models [13–17], making them potential vaccine candidates.

In a previous study, we identified a series of genes associated with *B. abortus* S2308 virulence using miniTn5 transposon mutagenesis (unpublished data). One mutant with the gene *BAB_RS22915* interrupted by miniTn5 showed highly attenuated virulence in BALB/c mice. *BAB_RS22915* encodes a putative lytic transglycosylase that is a homolog of membrane-bound lytic transglycosylase B (MltB). MltB cleaves the β-(1→4)-glycosidic bond between the *N*-acetylmuramic acid and *N*-acetylglucosamine residues of bacterial heteropolymer peptidoglycan [18]. In addition to bacterial cell wall recycling [19] and antibiotic resistance [20], MltB is also involved in assembly of macromolecular transport systems such as the type IV secretion system in Gram-negative bacteria [21]. We expected that deletion of *BAB_RS22915* would make the S2308 strain a good potential vaccine candidate; to investigate this, we generated the site-directed deletion mutant strain ∆22915. The virulence and protection capability of the mutant strain were evaluated. Our results demonstrated that the mutant strain ∆22915 was a highly attenuated strain and provided long-term, effective protection against wild-type, virulent strain S2308 challenge. These results suggested the mutant could be used as a novel vaccine candidate in the future.

## Materials and methods

### Ethics statement

This study was performed in strict accordance with the recommendations in the Guide for the Care and Use of Laboratory Animals of the Institutional Animal Care and Use Committee guidelines set by Shanghai Veterinary Research Institute, Chinese Academy of Agricultural Sciences (CAAS). BALB/c mice (SLAC, Experimental Animal Inc., Shanghai, China) were kept in cages and given water and food ad libitum under biosafety conditions. The protocol for animal experiments was approved by the Committee on the Ethics of Animal Experiments of Shanghai Veterinary Research Institute, CAAS (shvri-MO-0135).

### Bacterial strains, cell lines and plasmids

The virulent *B. abortus* S2308 strain was from American Type Culture Collection (ATCC, Manassas, VA, USA). Vaccine strain RB51 was kindly provided by Professor Qingming Wu from China Agriculture University, Beijing. Both strains were cultured in tryptic soy broth (TSB, Difco, Becton–Dickinson, Sparks, MD, USA) or tryptic soy agar (TSA) at 37 °C with 5% CO_2_. *B. abortus* S2308 with nalidixic acid resistance was induced with nalidixic acid at 50 μg/mL and preserved in our laboratory. *Escherichia coli* DH5α competent cells (Tiangen, Beijing, China) were cultured in Luria–Bertani (LB) media at 37 °C. Murine macrophage RAW 264.7 cells were from ATCC and cultured in Dulbecco’s modified Eagle medium (DMEM, Hyclone, GE Lifesciences, Logan, UT, USA) media with 10% fetal bovine serum (FBS, Gibco, ThermoScientific, Grand Island, NY, USA) at 37 °C with 5% CO_2_. The suicide pSC plasmid with the *sacB* gene [22] was preserved in our laboratory and used to construct a site-directed mutant strain.

### Construction of the mutant strain ∆22915

The ∆22915 strain was constructed as described previously [23]. Primers for construction were designed using the sequence of *BAB_RS22915* in the *B. abortus* S2308 genome (GenBank Code: NC_007618.1). Fragments that flanked *BAB_RS22915* were amplified in two independent PCR reactions using PrimeSTAR Max Mix (TaKaRa, Dalian, China) with primer pairs BAB_RS22915 UF/BAB_RS22915 UR, and BAB_RS22915 DF/BAB_RS22915 DR. Recovered PCR products were used for overlap PCR to produce joint sequences with primer pairs BAB_RS22915 UF/BAB_RS22915 DR. PCR products were purified, digested with *Xba*I and ligated into pSC. A recombinant plasmid with the correct sequence was designated pSC-∆22915 and introduced into DH5α.

Allelic replacement was employed to delete *BAB_RS22915* from the wild-type strain S2308. According to the method described previously [23], S2308 was cultured and collected by centrifugation at the exponential phase. After an ice bath for 15 min, S2308 was washed twice with ice-cold sterile water. Bacteria were resuspended in 10% (v/v) glycerin water and 3–5 μg recombinant pSC-∆22915 plasmid was added on ice. After electroporation, transformed S2308 were immediately transferred to prewarmed TSB media and cultured overnight. Bacteria were cultured on TSA plates with ampicillin at 100 μg/mL. A single exchanged mutant was selected and inoculated into TSB without antibiotics and cultured on TSA containing 5% (w/v) sucrose to produce a second exchange mutant. At least ten colonies per plate were collected for identification with PCR or quantitative real-time PCR (qRT-PCR). Primer pair BAB_RS22915 FF/BAB_RS22915 FR, flanking the gene coding sequence, and primer pair BAB_RS22915 OF/BAB_RS22915 OR, partially overlapping the deleted sequence, were used to identify gene deletions. Colonies with length-reduced fragment from BAB_RS22915 FF/BAB_RS22915 FR pair and no fragment from BAB_RS22915 OF/BAB_RS22915 OR pair were selected as *BAB_RS22915* deleted mutant. Primer pairs RT-22910 F/RT-22910 R, RT-22920 F/RT-22920 R and RT-22915 F/RT-22915 R were used for qRT-PCR to identify if BAB_RS22915 deletion had polar effects on flanking gene transcription. Deletion mutants were designated ∆22915. Primers and plasmids are listed in Table 1.

**Table 1:** **Strains, plasmids and primers used in this study**

Primers or plasmids	Description	Source or reference
Bacterial strains		
*B. abortus* S2308	Wild type strain; smooth phenotype	ATCC
RB51	Vaccine strain; rough phenotype	This study
∆22915	*BAB_RS22915* gene deletion mutant strain; smooth phenotype	This study
*Escherichia coli* (DH5α)	F^−^ φ80*lac*Z∆M15∆(*lac*ZYA-*arg*F)U169 *rec*A1 *end*A1 *hsd*R17(r_k_^−^, m_k_^+^) *pho*A *sup*E44 *thi*-1 *gyr*A96 *rel*A1 λ^−^	Tiangen
Plasmids		
pSC	Amp^R^; pUC19 plasmid containing *SacB* gene	[22]
Primers		
BAB_RS22915 UF	GCTCTAGACGTATATTCATCATCCGCAG (*Xba*I underlined)	This study
BAB_RS22915 UR	TGAGTTGATCCTGCGTCAGACTGAGGCGATAATCTTCATG	This study
BAB_RS22915 DF	CATGAAGATTATCGCCTCAGTCTGACGCAGGATCAACTCA	This study
BAB_RS22915 DR	GCTCTAGAGACATTGGAGGTGATTGCC (*Xba*I underlined)	This study
BAB_RS22915 FF	GCCACCCAACTTAGCGTGAG	This study
BAB_RS22915 FR	AAGTGGCGGCACCAAGAG	This study
BAB_RS22915 OF	GATGGCAAGGTCGATCTG	This study
BAB_RS22915 OR	GCCTGTCGAGAAGTTCCTG	This study
RT-22910 F	AAAGCACCGTTTTGCTCATC	This study
RT-22910 R	GCCAGACGGTTCATGTAGTG	This study
RT-22920 F	CCTCATCTGGAAAGTGCTGC	This study
RT-22920 R	CGAGAAAGAGTCCAAGCGTG	This study
RT-22915 F	ATAATGCCGTCAACATGCCG	This study
RT-22915 R	GGAAATGAGGCGCTTGGAAA	This study
RT-GAPDH F	GACATTCAGGTCGTCGCCATCA	[23]
RT-GAPDH R	TCTTCCTTCCACGGCAGTTCGG	[23]

^R^Antibiotic resistance.

### Extraction and silver staining of *Brucella* lipopolysaccharide (LPS)

Mutant strain ∆22915 and wild-type strain S2308 were cultured in TSB and collected at the exponential phase with centrifugation. Bacterial LPS was extracted with LPS Extraction Kits (iNtRON, Seoul, Korea). Samples were loaded on 12.5% polyacrylamide gels for SDS-PAGE and silver staining to validate LPS integrity. After electrophoresis, gels were fixed with periodic acid solution (0.3 M periodic acid, 40% v/v ethanol, 5% v/v acetic acid) at room temperature for 20 min. After washing with ultrapure water three times, gels were stained with silver–ammonia solution (0.02 M NaOH, 1.3% v/v ammonia water, 0.67% w/v AgNO_3_) at room temperature for 10 min. Gels were washed with ultrapure water to remove free Ag^+^ and incubated with coloring solution (0.005% w/v citric acid, 0.005% v/v formaldehyde) for 5–10 min. Reactions were halted with 10% (v/v) acetic acid solution and gels were imaged with an Odyssey Infrared Imaging System (LI-COR Biosciences, Lincoln, NE, USA).

### RNA extraction and quantitative real-time PCR

Strains ∆22915 and S2308 were cultured in TSB and collected at the exponential phase. Total RNA was extracted with TRIzol RNA isolation reagent (Ambion, Carlsbad, CA, USA). Genomic contamination was removed with Turbo DNA-free kits (Ambion). RNA was subjected to reverse transcription with PrimeScript RT reagent kits (TaKaRa) at 37 °C for 10 min, then 85 °C for 5 s for cDNA templates. GoTaq qPCR master mix (Promega, Fitchburg, WI, USA) was used for qRT-PCR, according to the manufacturer’s instruction: 1 μL cDNA, 0.5 μL forward or backward primer (10 μM), 8 μL nuclease-free water and 10 μL 2× GoTaq qPCR master mix were added. Reactions were on a Mastercycler ep Realplex system (Eppendorf, Germany) at 95 °C for 2 min, 40 cycles at 95 °C for 15 s, 60 °C for 1 min and a melting curve. Genes were tested in triplicate and the GAPDH gene was the internal control. Primers were designed according to the wild-type strain S2308 genome (GenBank Code: NC_007618.1 and NC_007624.1) with National Center for Biotechnology Information (NCBI) Primer-BLAST [24]. Relative transcription levels were calculated with the 2^−∆∆Ct^ method.

### Growth assays

Bacterial growth was measured at optical density 600 nm (OD_600_). Strains ∆22915 and S2308 were cultured in TSB for growth curves as described [22]. Freshly cultured bacteria were diluted to OD_600_ 1.0, then 1 mL was inoculated into 100 mL TSB and cultured at 37 °C at 200 rpm. OD_600_ absorbance of aliquots was measured every 4 h.

### Bacterial adherence, invasion and intracellular survival assays

Bacterial adherence, invasion and intracellular survival were tested using RAW 264.7 cells. Cells were seeded in 24-well plates (Corning, NY, USA) at 2 × 10^5^ per well and cultured in DMEM media with 10% FBS at 37 °C with 5% CO_2_. After 20 h, cells were washed twice with phosphate-buffered saline (PBS, Hyclone) and counted. Cells were infected with strain ∆22915 or S2308 at 100 multiplicity of infection (MOI). Plates were centrifuged at 400 × *g* for 5 min followed by 37 °C for 1 h. Cells were washed twice with PBS to remove nonadherent bacteria.

For adherence assays, wells of infected cells were incubated with 200 μL 0.2% (v/v) Triton X-100 (Sigma-Aldrich, St Louis, MO, USA) water solution for 10 min at 37 °C and 100 μL cell suspension was used for tenfold serial dilutions in PBS. Dilutions (100 μL) were spread on TSA plates and cultured at 37 °C with 5% CO_2_ for 72 h. For invasion assays, cells were treated with DMEM containing 100 μg/mL gentamicin for 1 h to kill extracellular bacteria after infection. Colony forming units (CFUs) were counted to determine adherent and invading bacteria. Invasion ratio was calculated as the number of invading bacteria versus the number of adherent bacteria.

For intracellular survival assays, after killing extracellular bacteria, cells were cultured in DMEM with 0.5% FBS and 50 μg/mL gentamicin. At 2, 8, 24 and 48 h post-infection (hpi), cells were washed and incubated with 200 μL 0.2% (v/v) Triton X-100 water solution for 10 min at 37 °C. Then, 100 μL of each dilution was collected to determine CFUs per well. Numbers of recovered bacteria at each time point were determined and compared with *B. abortus* S2308 to evaluate intracellular survival capacity of ∆22915.

### Immunofluorescence assays

RAW 264.7 cells were cultured on 15-mm glass diameter coverslips (Thermo Scientific, Waltham, MA, USA) in 24-well plates and infected with strain ∆22915 or S2308 at 100 MOI as described above. At 4 and 24 hpi, cells were washed twice with PBS and fixed overnight in 4% (w/v) paraformaldehyde at 4 °C. After three washes with PBS, cells were incubated with PBS containing 0.5% (v/v) Triton-X100 at room temperature for 10 min, followed by blocking with 5% (w/v) bovine serum albumin in PBS at 37 °C for 30 min. Cells were incubated with primary antibody diluted in 0.05% (v/v) Tween-20 PBS (PBST) for 45 min at 37 °C. After three washes with PBST, cells were incubated with secondary antibody for 45 min at 37 °C. After washing with PBST, coverslips were incubated with 4,6-diamidino-2-phenylindole at 2 μg/mL at room temperature. Coverslips were mounted on glass slides with Eukitt quick-hardening mounting medium (Sigma-Aldrich) and observed under laser scanning confocal microscope (Nikon D-Eclipse C1, Tokyo, Japan) with 100× oil immersion objective. Projections were saved in TIFF format and imported into Adobe Photoshop CS4 (Adobe Systems Incorporated, San Jose, CA, USA) to be merged. About 80–150 bacteria were counted randomly per coverslip and the percentage of lysosome-associated membrane protein 1 (LAMP-1) co-localized *Brucella*-containing vacuoles (BCV) was determined. Assays were performed in triplicate.

Rabbit anti-*Brucella* polyclonal antibody (1:500 dilution) was used to track intracellular bacteria. Rat LAMP-1 monoclonal antibody (1:1000 dilution, Abcam, USA) was used to track lysosomes. Goat anti-rabbit Alexa Fluor 488 and goat anti-rat Alexa Fluor 555 (Molecular Probes, Life Technologies, Eugene, OR, USA) were secondary antibodies at 1:1000 dilution.

### In vivo survival experiments

To investigate bacterial survival in vivo, strain S2308, strain ∆22915 and vaccine strain RB51 were intraperitoneally (IP) inoculated into 4- to 6-week-old female BALB/c mice (*n* = 6 per group) at 1 × 10^5^ CFU. Mice were euthanized at 2, 4, 6, 9 and 12 weeks post infection (wpi). Spleens were collected, weighed and homogenized in 5 mL 0.25% (v/v) Triton X-100 water solution and 100 μL aliquots were used for tenfold serial dilutions plated on TSA to determine bacterial CFUs. One mouse per group was euthanized and spleens and kidneys were collected and fixed in 4% (v/v) formaldehyde for histopathological examination. Peripheral blood samples of mice infected with ∆22915 or S2308 were collected. Levels of TNF-α and IL-12p40 in sera were detected with enzyme linked-immunosorbent assay (ELISA) (Yaoyun, Shanghai, China) to evaluate inflammation.

### Construction of *Brucella*-specific transcriptome library

A *Brucella* transcriptome library was constructed for strand-specific RNA deep sequencing at Beijing Genomics Institute. Strains ∆22915 and S2308 were cultured in TSB media and total RNA was extracted with RiboPure Bacteria Kits (Ambion) and ribosomal RNA removed with TruSeq RNA Sample Prep Kits v2 (Illumina, San Diego, CA, USA). After RNA was fragmented, first-strand cDNA was synthesized with First Strand Master Mix and Super Script II (Invitrogen, Carlsbad, CA, USA) with a program of 25 °C for 10 min, 42 °C for 50 min and 70 °C for 15 min. Product was purified with Agencourt RNAClean XP Beads (Beckman Coulter, Fullerton, CA, USA), then Second Master Mix (Invitrogen) and dATP, dGTP, dCTP, dUTP mix was added to synthesize second-strand cDNA. After end repair and A-tailing, purified product was treated with uracil-*N*-glycosylase. Then, the cDNA fragments were enriched with several rounds of PCR using Phusion High-Fidelity DNA polymerase (New England Biolabs, Beverly, MA, USA) and universal PCR primers.

The fragment distribution of the library was checked with an Agilent 2100 bioanalyzer instrument (Agilent Technologies, Santa Clara, CA, USA) and quantity checked by quantitative PCR. The qualified library was amplified to generate the cluster on the flowcell (TruSeq PE Cluster Kit V3-cBot-HS, Illumina). The amplified flowcell was used for pair-end sequencing on a HiSeq 2000 System (TruSeq SBS KIT-HS V3, Illumina) with read lengths of 90 bp. Acquired reads were mapped to the *B. abortus* S2308 genome (GenBank Accession: NC_007618.1 and NC_007624.1) and annotated gene sets obtained from NCBI Gene [25] using HISAT [26] and Bowtie [27] tools.

Reads that matched annotated genes were analyzed for expression differences between ∆22915 and S2308. Expression levels were determined using RSEM [28] software and calculated with a fragments per kilobase of transcript per million mapped reads (FPKM) algorithm [29]. Genes with FPKM >2.0-fold between the two strains were considered differentially expressed and validated with qRT-PCR using the protocol described above and primers in Additional file 1.

### Animal immunization assays

Female BALB/c mice (*n* = 5 per group) at 4–6 weeks were IP inoculated with ∆22915 or RB51 at 1 × 10^5^ CFU. Mice IP-inoculated with PBS were the blank controls. At 12 and 16 weeks post-vaccination (wpv), mice were challenged with 1 × 10^4^ CFU *B. abortus* S2308 that was nalidixic acid resistant. One week after challenge, mice were euthanized and spleens collected. As described above, spleens were homogenized in 5 mL 0.25% (v/v) Triton X-100 water solution. Each 100 μL of aliquot was serially tenfold diluted and spread on TSA with 30 μg/mL nalidixic acid to determine bacterial loads. Anti-*Brucella* ELISA titers in serum were detected at 2, 4, 6, 9 and 12 wpv as described previously [13], using heat-killed and sonicated *B. abortus* S2308 as coating antigen. The highest dilution with OD_450_ absorbance that was at least twice the mean value of the negative sample readings was used as the ELISA titer.

### Statistical analysis

CFU data from adherence, invasion and intracellular survival assays, in vivo persistence assay, and animal protection assay, as well as anti-*Brucella* ELISA titers from serum were converted to logarithmic numbers. Data were imported into GraphPad Prism 6 (Graph Pad Software, San Diego, CA, USA) for analysis. Statistical significance was determined using an unpaired or two-tailed Student’s *t* test. For group analysis, two-way ANOVA followed by Holm–Sidak’s multiple tests was used. *P* values less than 0.05 were considered statistically significant.

## Results

### Mutant strain ∆22915 was constructed without phenotype changes

**Figure 1:**
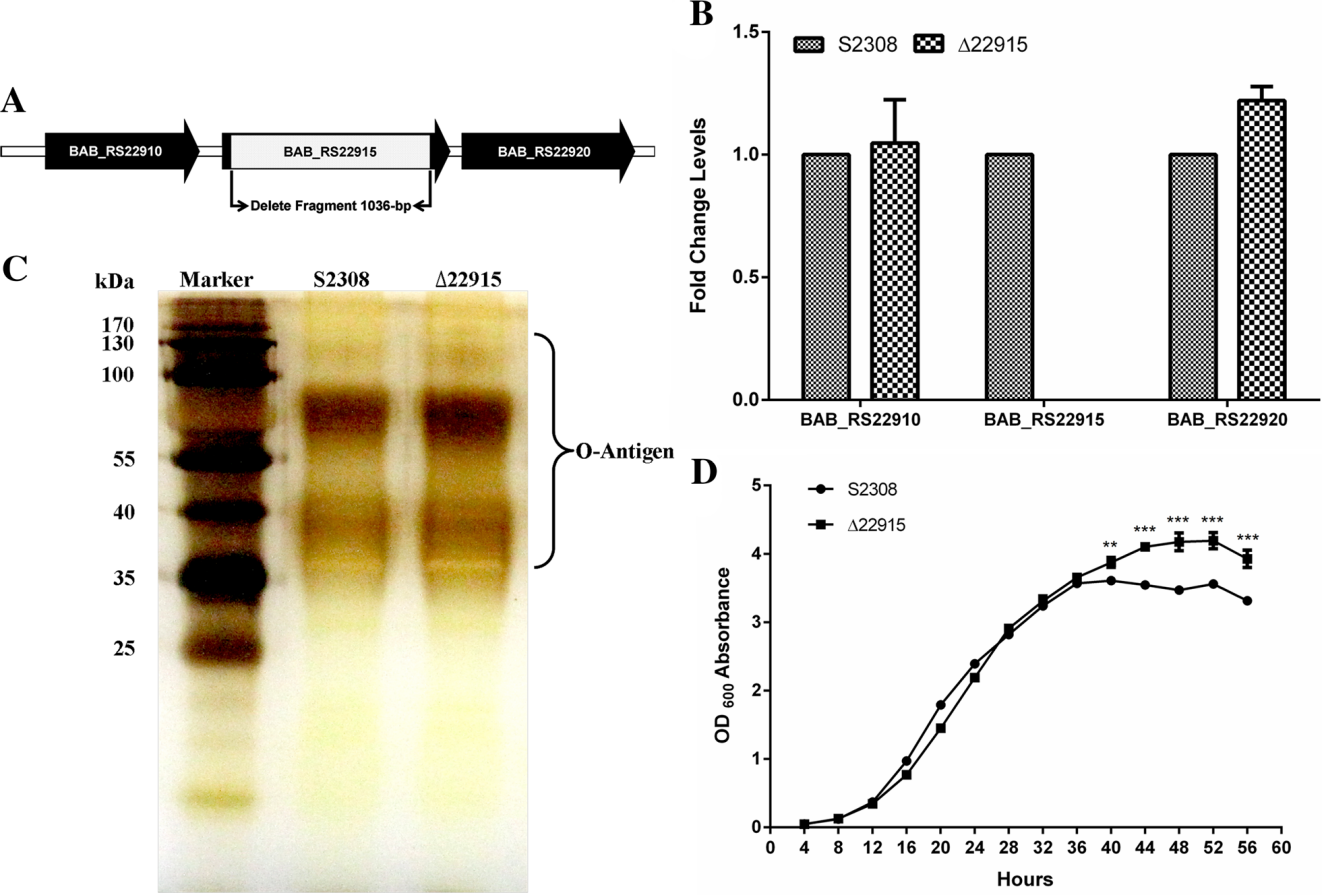
**Characterization of mutant strain** **∆22915. A** Schematic of *BAB_RS22915* gene deletion. A 1036-bp fragment was deleted from the *BAB_RS22915* coding sequence. **B** Transcription of *BAB_RS22915* and flanking genes in ∆22915. Transcription of *BAB_RS22915* was abolished and had no influence on transcriptional *BAB_RS22910* and *BAB_RS22920*. Data were presented as mean ± SD, and analyzed using a Student’s *t* test. **C** Silver staining of bacterial LPS. No difference was seen in LPS patterns between ∆22915 and wild-type strain S2308. Lane Marker: Prestain page ruler (Thermo Scientific, USA); Lane S2308: *B. abortus* S2308 LPS; Lane ∆22915: ∆22915 LPS. **D** Bacterial growth curves. Strains ∆22915 and S2308 were cultured in TSB media, and OD_600_ was measured every 4 h to monitor growth. Data were presented as mean ± SD, and analyzed with two-way ANOVA. **P < 0.01; ***P < 0.001.

A 1036-bp fragment was deleted from the *BAB_RS22915* coding gene sequence with a suicide plasmid (Figure 1A). qRT-PCR confirmed that *BAB_RS22915* gene expression was inactivated and did not influence flanking gene transcription (Figure 1B).

*Brucella* is reported to have a tendency to lose the O-antigen of LPS during mutant construction [30, 31]; this is a critical virulence factor for intracellular survival [32]. To identify if mutant strain ∆22915 had this spontaneous mutation, LPS purification and silver staining were performed. No LPS pattern changes were seen between strain ∆22915 and the wild-type strain S2308 (Figure 1C). Strain ∆22915 had a similar growth rate before 36 h when cultured in TSB, but a higher growth rate thereafter, compared with wild-type strain S2308 (Figure 1D), based on OD_600_. Bacterial CFU were 5 × 10^9^ CFU/mL for both strains at OD_600_ = 1.0.

### Strain ∆22915 showed reduced intracellular survival and failure to escape from lysosome fusion in RAW 264.7 cells

**Figure 2:**
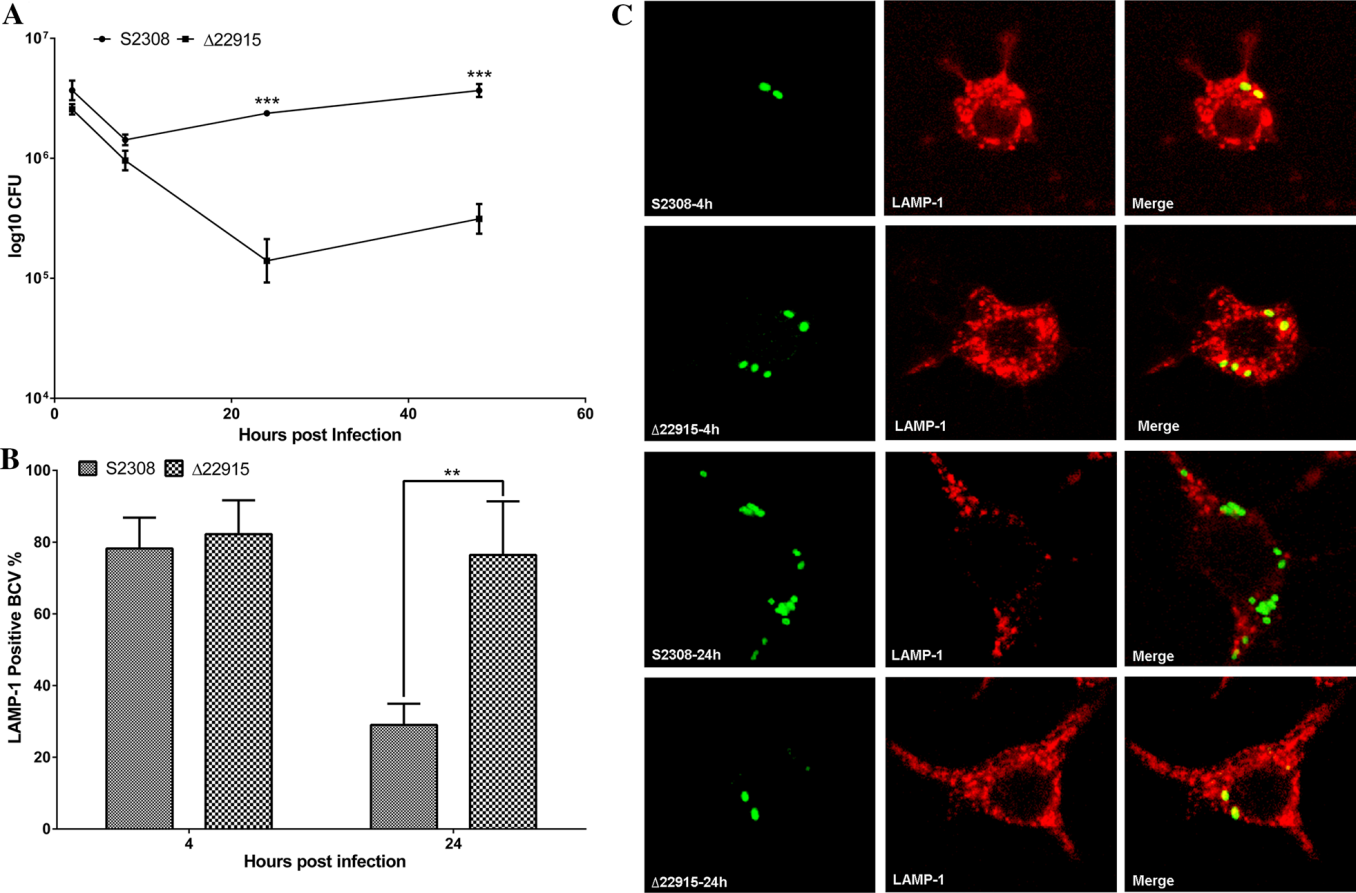
**Intracellular survival and traffic of mutant strain ∆22915 in RAW264.7 cells. A** Intracellular survival of ∆22915 was significantly decreased compared to wild-type strain S2308 at 24 and 48 h post infection. Data were presented as mean ± SD, and analyzed with two-way ANOVA. ***P < 0.001. **B** Determination of LAMP-1-positive BCVs in RAW264.7 cells. LAMP-1-positive BCV ratio was significantly higher for ∆22915-infected cells than S2308-infected cells at 24 h post infection. Data were presented as mean ± SD, and analyzed using a Student’s *t* test. **P < 0.01. **C** Representative images of LAMP-1-positive or -negative BCVs of RAW264.7 cells.

To evaluate if the *BAB_RS22915* gene was involved in *Brucella* invasion and intracellular survival, RAW 264.7 cells were infected with ∆22915 or S2308 at 100 MOI. Strain ∆22915 adhered to and invaded RAW264.7 cells as effectively as S2308. No significant difference was seen in adherence and invasion capacity between the mutant and wild-type strains (data not shown). However, the mutant strain showed reduced intracellular survival after 8 hpi; it was significantly reduced by more than tenfold at 24 hpi and thereafter, compared to S2308 (Figure 2A).

To determine if the intracellular survival defect of the mutant was associated with the capacity of BCVs to maturate or its capacity for intracellular trafficking, we determined the number of LAMP-1 positive BCV at 4 and 24 hpi. Strain ∆22915 failed to exclude LAMP-1 at 24 hpi, which might be the reason for the decreased intracellular survival (Figures 2B and C). Stress resistance assays showed no difference in resistance to low pH or H_2_O_2_ between ∆22915 and S2308 (data not shown).

### Strain ∆22915 is highly attenuated and induces no histopathological changes in mice

**Figure 3:**
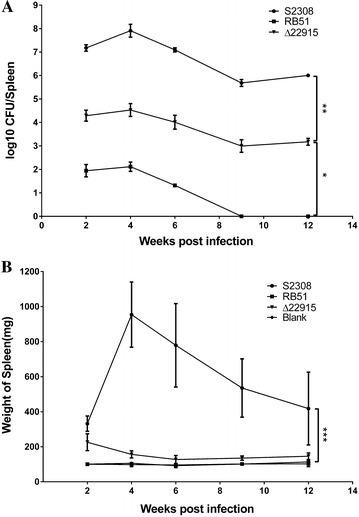
**Comparison of the persistence of mutant strain ∆22915, wild-type strain S2308 and vaccine strain RB51.** Five female BALB/c mice were IP-inoculated with ∆22915, S2308 or RB51 at 1 × 10^5^ CFU/mouse. **A** Bacterial loads in spleens were determined at 2, 4, 6, 9 and 12 weeks post infection. Persistence of ∆22915 in mice was significantly decreased compared to that of strain S2308 at all time points investigated. Vaccine strain RB51 was cleared at 9 wpi. **B** There was no significant difference in spleen weights among the uninfected mice, or mice infected with mutant strain ∆22915, or vaccine strain RB51. Spleen weights from S2308-infected mice were significantly increased. Data were presented as mean ± SD (*n* = 5), and analyzed with two-way ANOVA. *P < 0.05; **P < 0.01; ***P < 0.001.

To evaluate the virulence of mutant strain ∆22915, bacteria were IP inoculated into female BALB/c mice at 1.0 × 10^5^ CFU. Wild-type strain S2308 and vaccine strain RB51 were inoculated by the same route and dose as controls. Bacterial loads in spleens of ∆22915-infected mice were significantly reduced by around 1000-fold compared to S2308 infected mice at all time points investigated (Figure 3A). Splenomegaly was assessed by weighing spleens from infected mice. Spleen weight in wild-type-infected mice reached a peak at 4 wpi, then gradually decreased in the following weeks. Splenomegaly and spleen weight increases were not found in mice infected with ∆22915, which showed no significant differences from normal, uninfected spleens (Figure 3B). This result indicated the attenuated virulence of mutant strain ∆22915 in vivo.

**Figure 4:**
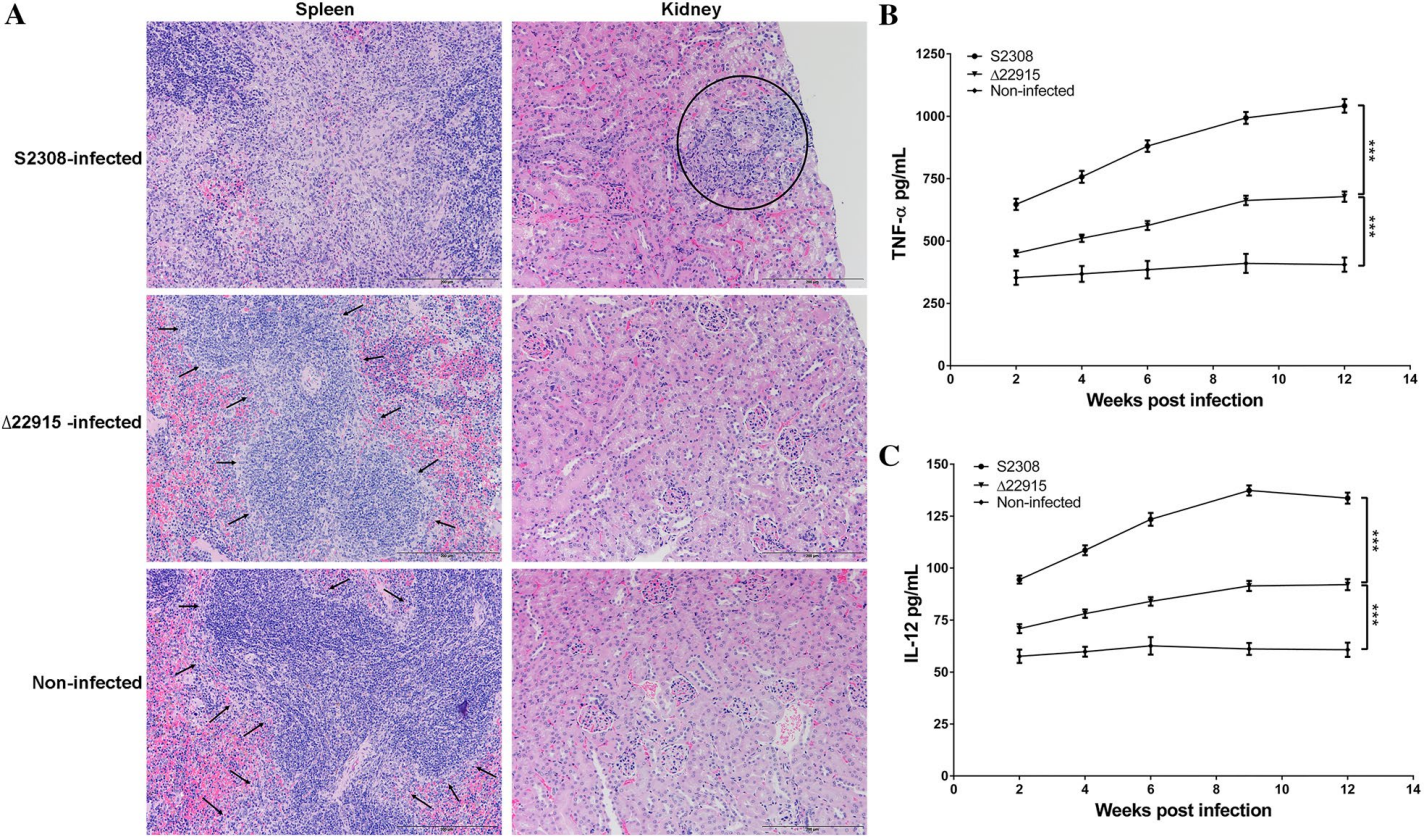
**Histopathological examination and proinflammatory cytokine determination. A** Histological examinations of spleen and kidney at 12 wpi from mice infected with mutant strain ∆22915 and wild-type strain S2308. Non-infected spleens and kidneys were used as normal controls. Strain ∆22915 caused no observable pathological damage in spleens or kidneys. Arrows indicate normal boundaries between red and white pulps in ∆22915-infected spleen, which was similar with uninfected spleen, however, no clear boundary was shown in strain S2308 infected spleen due to tissue damage. Kidney lesions caused by strain S2308 infection are circled. Bars represent 200 μm. **B** Determination of TNF-α in peripheral blood. TNF-α induction by ∆22915 was significantly lower than by S2308, but higher than by no infection. **C** Determination of IL-12p40 in peripheral blood. IL-12p40 induction by ∆22915 was significantly lower than by S2308 but higher than by no infection. Data were expressed as mean ± SD (*n* = 5) and analyzed with two-way ANOVA. ***P < 0.001.

Histopathological examination at 12 wpi showed that strain ∆22915 caused no observable pathological lesions (Figure 4A). In spleen of ∆22915-infected mouse, the boundary of red and white pulps was clear (indicated by arrows), and no reticular tissue proliferation or inflammatory cell infiltration was observed. On the other hand, wild-type strain S2308 caused extensive proliferation of reticular tissue and necrosis of mature lymphocytes in spleens, seen as an unclear boundary of red and white pulps. In kidney of ∆22915-infected mouse, the structure of renal tubules was intact. However, severe basophil infiltration, epithelial cell necrosis and atrophy was observed in renal tubules in S2308-infected mouse kidney. No observable lesions were found in organs of uninfected mice.

The production of proinflammatory cytokines TNF-α and IL-12p40 in peripheral blood was determined (Figures  4B and C). Strain ∆22915 induced significant less cytokines at all time points investigated, compared to strain S2308. However, the cytokine levels induced by strain ∆22915 were much higher than those in the normal, noninfected mice (*P* < 0.001). These results demonstrated that the virulence of the mutant strain ∆22915 was attenuated, facilitating its application as a novel vaccine candidate.

### Transcriptomic analysis

**Figure 5:**
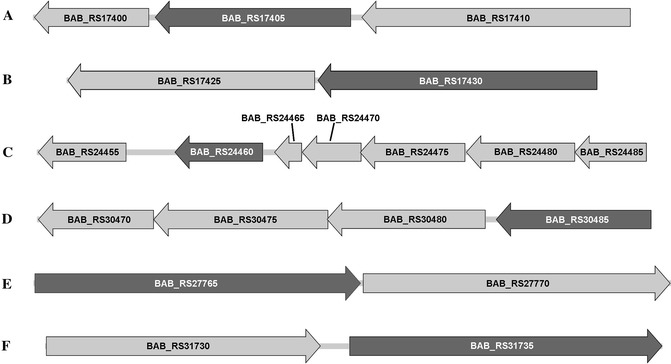
**Genetic organization of upregulated gene locus. A** *BAB_RS17405* encodes a dihydropyrimidinase on chromosome I of *B. abortus* S2308. It is flanked by a zinc-dependent allantoate amidohydrolase (*BAB_RS17410*) and a dihydrorhizobitoxine desaturase (*BAB_RS17400*). **B** *BAB_RS17430* encodes the α-subunit of NADPH dependent glutamate synthase on chromosome I of *B. abortus* S2308, downstream of β-subunit B of NADPH dependent glutamate synthase (*BAB_RS17425*). **C** *BAB_RS24460* encodes a substrate-binding protein on chromosome I of *B. abortus* S2308. It is flanked by genes for two hypothetical proteins without complete coding sequences (*BAB_RS24465* and *24470*), an ATP-binding protein with ATPase enzymatic activity (*BAB_RS24475*), two permease proteins (*BAB_RS24480* and *24485*) and another substrate-binding protein (*BAB_RS24455*). **D** *BAB_RS30485* encodes a substrate binding protein which is located on chromosome II of *B. abortus* S2308. It is flanked by two permease proteins (*BAB_RS30470* and *30475*) and another ATP-binding protein (*BAB_RS30480*). Both ABC transporters are predicted to be involved in amino acid transport and metabolism. **E** *BAB_RS27765* encodes a putative fumarylacetoacetate (Faa) hydrolase without known function. It is on chromosome II of *B. abortus* S2308, upstream of a gene for galactose 1-dehydrogenase (*BAB_RS27770*). **F** *BAB_RS31735* encodes a putative amidohydrolase without known function. It is on chromosome II of *B. abortus* S2308, downstream of a gamma-glutamyl-gamma-aminobutyraldehyde dehydrogenase (*BAB_RS31730*) gene. Arrows indicate direction of CDS. Except *BAB_RS27765* and *31735*, all other CDSs are on the complementary strand of the *B. abortus* S2308 genome. Tags of upregulated genes are indicated by a darker color.

The transcriptome of the mutant strain ∆22915 was compared with that of the wild-type strain S2308 using strand-specific RNA-Seq analysis. In total, 14 399 044 reads were acquired. The average mapping ratio was 97.38% for the reference genome and 78.94% for the reference genes. Analysis of the gene-matched reads revealed that 16 genes had a high probability of being dis-transcript by more than twofold in the mutant strain compared to the wild-type strain. qRT-PCR indicated that the transcription of six genes was upregulated, and four of them were upregulated more than tenfold (Table 2). EggNOG 4.5 [33] analysis showed that four genes were categorized as “amino acid/nucleotide transport and metabolism” and the other two had no designated functions. The product of the *BAB_RS17405* gene was involved with “nucleotide transport and metabolism” (Figure 5A). *BAB_RS17430* encoded a NADPH-dependent glutamate synthase (Figure 5B). *BAB_RS24460* and *BAB_RS30485* encoded two substrate-binding proteins of two amino acid ABC transporters (Figures 5C and D). *BAB_RS27765* encoded a hypothetical protein without known function (Figure 5E). *BAB_RS31735* encoded a putative amidohydrolase without known function (Figure 5F). These results indicated that the expression of amino acids/nucleotides transport and metabolism related protein was enhanced in the mutant strain ∆22915.

**Table 2:** **Real-time PCR verification of differentially expressed genes in mutant strain** **∆22915**

Gene locus^a^	Description of genes	Function^b^	Fold changes (2^−∆∆Ct^) ±SD
*BAB_RS24460*	Extracellular ligand-binding receptor	Amino acid transport and metabolism	39.08 ± 4.25
*BAB_RS17405*	Dihydropyrimidinase	Nucleotide transport and metabolism	20.50 ± 2.27
*BAB_RS30485*	Extracellular solute-binding protein family 1	Amino acid transport and metabolism	15.35 ± 1.80
*BAB_RS17430*	Oxidoreductase	Amino acid transport and metabolism	12.67 ± 1.25
*BAB_RS31735*	Amidohydrolase	Function unknown	3.63 ± 0.27
*BAB_RS27765*	Fumarylacetoacetate (Faa) hydrolase	Function unknown	2.80 ± 0.22
*BAB_RS30280*	Quinone oxidoreductase	Energy production and conversion	1.82 ± 0.20
*BAB_RS30270*	Abc transporter permease protein	Amino acid transport and metabolism	1.70 ± 0.24
*BAB_RS26970*	Flagellar basal-body rod protein	Cell motility	1.62 ± 0.31
*BAB_RS18915*	Gene transfer agent	Function unknown	1.55 ± 0.22
*BAB_RS30285*	Transcriptional regulator, GntR family	Transcription	1.52 ± 0.26
*BAB_RS30275*	Extracellular ligand-binding receptor	Amino acid transport and metabolism	1.37 ± 0.14
*BAB_RS28745*	Abc transporter permease protein	Amino acid transport and metabolism	1.37 ± 0.35
*BAB_RS 28215*	Transposase	Replication, recombination and repair	1.25 ± 0.23
*BAB_RS27910*	Transcriptional regulator	Transcription	0.81 ± 0.19
*BAB_RS22920*	Auxin efflux carrier	Function unknown	0.76 ± 0.11

Genes with over twofold changes levels and high probability were further validated with qRT-PCR.

^a^Based on *B. abortus* S2308 genome (GenBank Code: NC_007618.1 and NC_007624.1).

^b^The functional categories of protein were predicted by searching through EggNOG database [33] with BLASTP.

### Strain ∆22915 induces immune responses and protects against S2308 challenge

**Figure 6:**
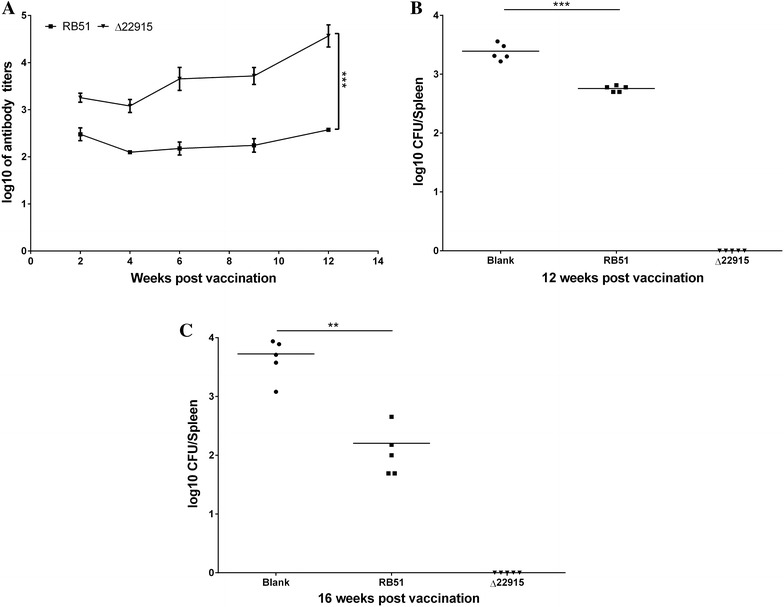
**Determination of** ***Brucella*** **antibody and protection by vaccination with mutant strain ∆22915. A** Strain ∆22915 induced significantly higher titers of *Brucella*-specific antibodies than RB51 at all investigated time points. Data were expressed as mean ± SD (*n* = 5) and analyzed with two-way ANOVA. ***P < 0.001. **B** Protection efficacy at 12 weeks post vaccination. **C** Protection efficacy at 16 weeks post vaccination. For protection evaluation, vaccinated mice were challenged with 1 × 10^4^ CFU nalidixic-acid resistant S2308/mouse. One week post challenge, spleens were collected to determine bacterial loads to assess protection efficacy. No bacteria were recovered from the mice of ∆22915-vaccinated group, indicating better protection efficacy than RB51. Data points were individual values of CFU determinations (*n* = 5) and analyzed using a Student’s *t* test. **P < 0.01; ***P < 0.001.

After vaccination with the mutant strain ∆22915, *Brucella* antibodies in sera were measured using ELISA at 2, 4, 6, 9 and 12 wpv. Antibody was induced as early as 2 wpv, and reached a peak at 12 wpv (Figure 6A). Strain ∆22915 induced higher antibody titers than the vaccine strain RB51, suggesting that ∆22915 effectively activated host humoral immunity.

Protection of mice against challenge by wild-type strain S2308 was investigated. Mice vaccinated with strain ∆22915 were challenged with nalidixic-acid resistant S2308 at 1.0 × 10^4^ CFU at 12 and 16 wpv. Mice vaccinated with strain RB51 and nonvaccinated mice were used as positive and negative protection controls, respectively. Spleens were collected at 1 week after challenge to determine bacterial loads. Strain ∆22915 provided better protection against S2308 challenge than vaccine strain RB51 (Figures 6B and C). No CFU was seen for S2308 in the ∆22915 vaccinated mice. More than 570 CFU/spleen were seen for S2308 counted in RB51-vaccinated mice. Bacterial loads in the nonvaccinated mice were 5290 CFU/spleen. This result showed that the mutant strain ∆22915 provided BALB/c mice with better protection against S2308 than RB51.

## Discussion

We successfully constructed the *B. abortus* mutant strain ∆22915 by deleting a 1036-bp fragment from the *BAB_RS22915* gene. Strain ∆22915 showed similar LPS phenotypes and adherence and invasion capacities compared to the wild-type strain S2308, but attenuated virulence, determined by in vivo and in vitro survival. We demonstrated that the decreased intracellular survival of strain ∆22915 was associated with the altered capacity to exclude lysosomes, an important step before *Brucella* reaches a replicative niche in endoplasmic reticulum [34]. This result suggested that the altered intracellular traffic contributed to decreased survival in RAW 264.7 cells.

MltB is a member of the lytic transglycosylase (LT) family that is involved in recycling bacterial cell walls to produce 1,6-anhydromuropeptides for bacterial growth [35]. However, inactivation of LTs does not inhibit bacterial growth in medium [36]. This finding indicates involvement of other pathways or gene upregulation to compensate for bacterial cell growth [37]. Thus, we investigated the gene expression in the whole genome using transcriptomic analysis, which indicated 16 genes were changed for their expression. qRT-PCR confirmed that four genes were upregulated by more than 10-fold; these were categorized into “amino acid/nucleotide transport and metabolism”. Transcription of *BAB_RS24460* was upregulated 39-fold in the mutant strain ∆22915; this is the substrate-binding component of the branched-chain amino acid ABC transporter, responsible for the uptake of leucine, isoleucine and valine [38]; uptake mediated by this transporter contributes to bacterial growth [39], intracellular survival [40], and symbiosis between host and bacteria [41]. The *BAB_RS30485* gene, also known as the *PotD* gene, is the substrate-binding component of the polyamine ABC transporter that preferentially takes up spermidine [42]. *BAB_RS17430* encodes the α subunit of the NADPH dependent glutamate synthase that catalyzes the reductive transfer of the amide group of l-glutamine to C-2 of 2-oxoglutarate to produce l-glutamate [43]. This reaction is involved in nitrogen assimilation of *α*-*proteobacteria* to produce glutamine [44]. *BAB_RS17405* encodes a dihydropyrimidinase that is responsible for the second step of pyrimidine reductive catabolism to produce *N*-carbamoyl-b-alanine and *N*-carbamoyl-b-aminoisobutyric acid, respectively, from dihydrouracil and dihydrothymine [45]. To some bacteria, it functions as an important source of nitrogen [46]. We found that upregulated genes are mainly responsible for bacterial metabolism. Therefore, they might have contributed to the enhanced growth rate of strain ∆22915 in stationary stage cultures in TSB media. The upregulated genes might be related to the decreased intracellular survival of the mutant strain ∆22915. Bacterial ABC transporters and metabolism-related proteins are reported to provide animals with protection as vaccine candidates [47–49]. We assume the upregulation of these genes may enhance the antigenicity of ∆22915 to activate the immune response of macrophages, resulting in its decreased intracellular survival.

In vivo experiments indicated that strain ∆22915 persisted in mice for longer than 12 weeks, but caused no observable pathological damage. In addition, the mutant strain ∆22915 induced fewer inflammatory responses than the wild-type strain. In previous research, live attenuated *Brucella* with multiple disregulated genes protected against the wild-type strain S2308 [50]. This result suggested that testing whether mutant strain ∆22915 could be applied as a vaccine would be a worthwhile study. Vaccination with the mutant strain induced an effective immune response against the wild-type strain S2308. After vaccination with ∆22915, bacterial loads decreased until after 4 wpv, with specific antibody titers increasing to a peak at 12 wpv. The smooth type of the mutant strain ∆22915 induced higher levels of antibody than RB51, due to the dominant antigenicity of the O-antigen [51]. The adaptive cellular response is mainly responsible for the immunity against *Brucella*, but antibody against O-antigen or serum from smooth *Brucella*-infected animals participates in defense against challenge by wild-type *Brucella* [52]. In challenge assays, coinciding with the humoral response, strain ∆22915 provided longer and better protection than RB51 at 12 and 16 wpv. Unlike *virB* mutant *Brucella* [53], challenge by S2308 did not rescue the survival of ∆22915 (data not shown), confirming its safety as a vaccine candidate.

In conclusion, using a suicide plasmid, we constructed a smooth-phenotype mutant strain ∆22915 with a deletion of the *BAB_RS22915* gene. In addition to altered intracellular traffic and attenuated survival, multiple genes involved in amino acid/nucleotide transport and metabolism were upregulated in the mutant strain. These genes may be associated with the attenuation of intracellular survival and require further research on their mechanism. Virulence of the mutant strain ∆22915 was significantly attenuated in BALB/c mice and provided better protection against *B. abortus* S2308 than RB51. This finding facilitated potential use of mutant strain ∆22915 as a novel vaccine candidate in the future.

## Additional file

**Additional file 1.** **Primers used for qRT-PCR validation.** ^a^ Based on *B. abortus* S2308 genome (GenBank Code: NC_007618.1 and NC_007624.1).

## References

[1] Pandey A, Cabello A, Akoolo L, Rice-Ficht A, Arenas-Gamboa A, McMurray D, Ficht TA, de Figueiredo P (2016). The case for live attenuated vaccines against the neglected zoonotic diseases brucellosis and bovine tuberculosis. PLoS Negl Trop Dis.

[2] Pappas G, Papadimitriou P, Christou L, Akritidis N (2006). Future trends in human brucellosis treatment. Expert Opin Investig Drugs.

[3] Pappas G (2010). The changing Brucella ecology: novel reservoirs, new threats. Int J Antimicrob Agents.

[4] Olsen SC, Stoffregen WS (2005). Essential role of vaccines in brucellosis control and eradication programs for livestock. Expert Rev Vaccines.

[5] Fluegel Dougherty AM, Cornish TE, O’Toole D, Boerger-Fields AM, Henderson OL, Mills KW (2013). Abortion and premature birth in cattle following vaccination with *Brucella abortus* strain RB51. J Vet Diagn Invest.

[6] Ollé-Goig JE, Canela-Soler J (1987). An outbreak of *Brucella melitensis* infection by airborne transmission among laboratory workers. Am J Public Health.

[7] Lin J, Ficht TA (1995). Protein synthesis in *Brucella abortus* induced during macrophage infection. Infect Immun.

[8] Truong QL, Cho Y, Barate AK, Kim S, Hahn TW (2014). Characterization and protective property of *Brucella abortus* cydC and looP mutants. Clin Vaccine Immunol.

[9] Truong QL, Cho Y, Barate AK, Kim S, Watarai M, Hahn TW (2015). Mutation of purD and purF genes further attenuates *Brucella abortus* strain RB51. Microb Pathog.

[10] Loisel-Meyer S, Jimenez de Bagues MP, Basseres E, Dornand J, Kohler S, Liautard JP, Jubier-Maurin V (2006). Requirement of norD for *Brucella suis* virulence in a murine model of in vitro and in vivo infection. Infect Immun.

[11] Kim S, Watanabe K, Shirahata T, Watarai M (2004). Zinc uptake system (znuA locus) of *Brucella abortus* is essential for intracellular survival and virulence in mice. J Vet Med Sci.

[12] Kim HS, Caswell CC, Foreman R, Roop RM, Crosson S (2013). The *Brucella abortus* general stress response system regulates chronic mammalian infection and is controlled by phosphorylation and proteolysis. J Biol Chem.

[13] Zhang M, Han X, Liu H, Tian M, Ding C, Song J, Sun X, Liu Z, Yu S (2013). Inactivation of the ABC transporter ATPase gene in *Brucella abortus* strain 2308 attenuated the virulence of the bacteria. Vet Microbiol.

[14] Kahl-McDonagh MM, Ficht TA (2006). Evaluation of protection afforded by *Brucella abortus* and *Brucella melitensis* unmarked deletion mutants exhibiting different rates of clearance in BALB/c mice. Infect Immun.

[15] Yang X, Clapp B, Thornburg T, Hoffman C, Pascual DW (2016). Vaccination with a DnorD DznuA *Brucella abortus* mutant confers potent protection against virulent challenge. Vaccine.

[16] Willett JW, Herrou J, Czyz DM, Cheng JX, Crosson S (2016). *Brucella abortus* DrpoE1 confers protective immunity against wild type challenge in a mouse model of brucellosis. Vaccine.

[17] Truong QL, Cho Y, Park S, Kim K, Hahn TW (2016). *Brucella abortus* DcydCDcydD and DcydCDpurD double-mutants are highly attenuated and confer long-term protective immunity against virulent *Brucella abortus*. Vaccine.

[18] Reid CW, Legaree BA, Clarke AJ (2007). Role of Ser216 in the mechanism of action of membrane-bound lytic transglycosylase B: further evidence for substrate-assisted catalysis. FEBS Lett.

[19] Suvorov M, Lee M, Hesek D, Boggess B, Mobashery S (2008). Lytic transglycosylase MltB of *Escherichia coli* and its role in recycling of peptidoglycan strands of bacterial cell wall. J Am Chem Soc.

[20] Lamers RP, Nguyen UT, Nguyen Y, Buensuceso RN, Burrows LL (2015). Loss of membrane-bound lytic transglycosylases increases outer membrane permeability and beta-lactam sensitivity in *Pseudomonas aeruginosa*. Microbiologyopen.

[21] Koraimann G (2003). Lytic transglycosylases in macromolecular transport systems of Gram-negative bacteria. Cell Mol Life Sci.

[22] Gao J, Tian M, Bao Y, Li P, Liu J, Ding C, Wang S, Li T, Yu S (2016). Pyruvate kinase is necessary for *Brucella abortus* full virulence in BALB/c mouse. Vet Res.

[23] Tian M, Qu J, Han X, Ding C, Wang S, Peng D, Yu S (2014). Mechanism of Asp24 upregulation in *Brucella abortus* rough mutant with a disrupted O-antigen export system and effect of Asp24 in bacterial intracellular survival. Infect Immun.

[24] Ye J, Coulouris G, Zaretskaya I, Cutcutache I, Rozen S, Madden TL (2012). Primer-BLAST: a tool to design target-specific primers for polymerase chain reaction. BMC Bioinform.

[25] National Center for Biotechnology Information Gene. http://www.ncbi.nlm.nih.gov/gene. Accessed 6 Sep 2016

[26] Kim D, Langmead B, Salzberg SL (2015). HISAT: a fast spliced aligner with low memory requirements. Nat Methods.

[27] Langmead B, Trapnell C, Pop M, Salzberg SL (2009). Ultrafast and memory-efficient alignment of short DNA sequences to the human genome. Genome Biol.

[28] Li B, Dewey CN (2011). RSEM: accurate transcript quantification from RNA-Seq data with or without a reference genome. BMC Bioinform.

[29] Mortazavi A, Williams BA, McCue K, Schaeffer L, Wold B (2008). Mapping and quantifying mammalian transcriptomes by RNA-Seq. Nat Methods.

[30] Braun W (1946). Dissociation in *Brucella abortus*; a demonstration of the role of inherent and environmental factors in bacterial variation. J Bacteriol.

[31] Pei J, Kahl-McDonagh M, Ficht TA (2014). Brucella dissociation is essential for macrophage egress and bacterial dissemination. Front Cell Infect Microbiol.

[32] Gonzalez D, Grillo MJ, De Miguel MJ, Ali T, Arce-Gorvel V, Delrue RM, Conde-Alvarez R, Munoz P, Lopez-Goni I, Iriarte M, Marin CM, Weintraub A, Widmalm G, Zygmunt M, Letesson JJ, Gorvel JP, Blasco JM, Moriyon I (2008). Brucellosis vaccines: assessment of *Brucella melitensis* lipopolysaccharide rough mutants defective in core and *O*-polysaccharide synthesis and export. PLoS One.

[33] Huerta-Cepas J, Szklarczyk D, Forslund K, Cook H, Heller D, Walter MC, Rattei T, Mende DR, Sunagawa S, Kuhn M, Jensen LJ, von Mering C, Bork P (2016). eggNOG 4.5: a hierarchical orthology framework with improved functional annotations for eukaryotic, prokaryotic and viral sequences. Nucleic Acids Res.

[34] von Bargen K, Gorvel JP, Salcedo SP (2012). Internal affairs: investigating the Brucella intracellular lifestyle. FEMS Microbiol Rev.

[35] Goodell EW (1985). Recycling of murein by *Escherichia coli*. J Bacteriol.

[36] Kraft AR, Prabhu J, Ursinus A, Holtje JV (1999). Interference with murein turnover has no effect on growth but reduces β-lactamase induction in *Escherichia coli*. J Bacteriol.

[37] Vollmer W, Holtje JV (2001). Morphogenesis of *Escherichia coli*. Curr Opin Microbiol.

[38] Trakhanov S, Vyas NK, Luecke H, Kristensen DM, Ma J, Quiocho FA (2005). Ligand-free and -bound structures of the binding protein (LivJ) of the *Escherichia coli* ABC leucine/isoleucine/valine transport system: trajectory and dynamics of the interdomain rotation and ligand specificity. Biochemistry.

[39] Nikodinovic-Runic J, Flanagan M, Hume AR, Cagney G, O’Connor KE (2009). Analysis of the Pseudomonas putida CA-3 proteome during growth on styrene under nitrogen-limiting and non-limiting conditions. Microbiology.

[40] Gesbert G, Ramond E, Tros F, Dairou J, Frapy E, Barel M, Charbit A (2015). Importance of branched-chain amino acid utilization in Francisella intracellular adaptation. Infect Immun.

[41] Prell J, White JP, Bourdes A, Bunnewell S, Bongaerts RJ, Poole PS (2009). Legumes regulate Rhizobium bacteroid development and persistence by the supply of branched-chain amino acids. Proc Natl Acad Sci U S A.

[42] Furuchi T, Kashiwagi K, Kobayashi H, Igarashi K (1991). Characteristics of the gene for a spermidine and putrescine transport system that maps at 15 min on the *Escherichia coli* chromosome. J Biol Chem.

[43] Vanoni MA, Curti B (1999). Glutamate synthase: a complex iron–sulfur flavoprotein. Cell Mol Life Sci.

[44] Ronneau S, Moussa S, Barbier T, Conde-Alvarez R, Zuniga-Ripa A, Moriyon I, Letesson JJ (2016). Brucella, nitrogen and virulence. Crit Rev Microbiol.

[45] Wallach DP, Grisolia S (1957). The purification and properties of hydropyrimidine hydrase. J Biol Chem.

[46] Kim S, West TP (1991). Pyrimidine catabolism in *Pseudomonas aeruginosa*. FEMS Microbiol Lett.

[47] Biswas S, Biswas I (2013). SmbFT, a putative ABC transporter complex, confers protection against the lantibiotic Smb in Streptococci. J Bacteriol.

[48] Riquelme-Neira R, Retamal-Diaz A, Acuna F, Riquelme P, Rivera A, Saez D, Onate A (2013). Protective effect of a DNA vaccine containing an open reading frame with homology to an ABC-type transporter present in the genomic island 3 of *Brucella abortus* in BALB/c mice. Vaccine.

[49] Nol P, Olsen SC, Rhyan JC, Sriranganathan N, McCollum MP, Hennager SG, Pavuk AA, Sprino PJ, Boyle SM, Berrier RJ, Salman MD (2016). Vaccination of elk (*Cervus canadensis*) with *Brucella abortus* strain RB51 overexpressing superoxide dismutase and glycosyltransferase genes does not induce adequate protection against experimental *Brucella abortus* challenge. Front Cell Infect Microbiol.

[50] Lei S, Zhong Z, Ke Y, Yang M, Xu X, Ren H, An C, Yuan J, Yu J, Xu J, Qiu Y, Shi Y, Wang Y, Peng G, Chen Z (2015). Deletion of the small RNA chaperone protein Hfq down regulates genes related to virulence and confers protection against wild-type *Brucella* challenge in mice. Front Microbiol.

[51] Ganesh NV, Sadowska JM, Sarkar S, Howells L, McGiven J, Bundle DR (2014). Molecular recognition of *Brucella* A and M antigens dissected by synthetic oligosaccharide glycoconjugates leads to a disaccharide diagnostic for brucellosis. J Am Chem Soc.

[52] Montaraz JA, Winter AJ, Hunter DM, Sowa BA, Wu AM, Adams LG (1986). Protection against *Brucella abortus* in mice with *O*-polysaccharide-specific monoclonal antibodies. Infect Immun.

[53] Nijskens C, Copin R, De Bolle X, Letesson JJ (2008). Intracellular rescuing of a *B. melitensis* 16 M virB mutant by co-infection with a wild type strain. Microb Pathog.

