# Supplementary material for: Characterization of Brucella abortus mutant strain Δ22915, a potential vaccine candidate
Source: Vet Res. 2017 Apr 4;48:17. doi: 10.1186/s13567-017-0422-9 (PMC5381064; doi:10.1186/s13567-017-0422-9)
Supplement: Supplementary file 1 — Additional file 1. Primers used for qRT-PCR validation. a Based on B. abortus S2308 genome (GenBank Code: NC_007618.1 and NC_007624.1). [file 13567_2017_422_MOESM1_ESM.doc]

**Additional file 1 Primers used for qRT-PCR validation.**

| **Primers**^a^ | **Description** | **Source or reference** |
| --- | --- | --- |
| RT-24460 F | GGCGTCAAGTTCGTGATTGG | This study |
| RT-24460 R | GACCGTAAGGGGTCTTGTCG | This study |
| RT-17405 F | ATGAGGTCATCGACGCATCC | This study |
| RT-17405 R | CAGGACGAAATCGACCACCA | This study |
| RT-30485 F | AAATTCCTCGACAGTGCGGT | This study |
| RT-30485 R | ATCCTTGTCGACGCCATCAG | This study |
| RT-17430 F | CTACGCCACCGATATGCTGA | This study |
| RT-17430 R | GGGCGAAATTGTTCACCGTC | This study |
| RT-31735 F | CTCGCTTGCCAAACTCGATG | This study |
| RT-31735 R | GCAACACGCTCGACAATACC | This study |
| RT-27765 F | GCAGAAAAGCCGACCGATTC | This study |
| RT-27765 R | GTCTTCAGCAAAAGCCGGTG | This study |
| RT-30280 F | AGGCGTAACGGATGTCTTGG | This study |
| RT-30280 R | CTGCGCGCTGAAAGAGATTG | This study |
| RT-30270 F | CTTCTGAACGGCCTGATGCT | This study |
| RT-30270 R | AACCGCCGCATAAGGATGAA | This study |
| RT-26970 F | CTTTCCTCGCTCATCACGCT | This study |
| RT-26970 R | TATAGCTCTTGCCCGCTGTG | This study |
| RT-18915 F | ATGGGCGGTTATGCCATTGA | This study |
| RT-18915 R | GTGGTGGTTTTGCGCTCTTC | This study |
| RT-30285 F | GGACGCACTCGAACAAATCC | This study |
| RT-30285 R | GGAATGCCTGCGTCGTAAAG | This study |
| RT-30275 F | AGTCGACATGTCTTCGCTGG | This study |
| RT-30275 R | GATCAGGTCATGTCCCACGG | This study |
| RT-28745 F | ATACTCGACCACTTCACGCC | This study |
| RT-28745 R | CAGAAGCGTGAAAACCGCAA | This study |
| RT-28215 F | GTCGATGCTATCGGCCTACC | This study |
| RT-28215 R | TTGATCTGAGCCGTTGCCTT | This study |
| RT-27910 F | GACTTGCCGATGGTGTTCCT | This study |
| RT-27910 R | AGACACGTTCGATGAGCAGG | This study |
| RT-22920 F | AACGCCGCCTATCCCAATAC | This study |
| RT-22920 R | AGCGGCTTCTTTTCGGTTTG | This study |

^a^ Based on *B. abortus* S2308 genome (GenBank Code: NC_007618.1 and NC_007624.1).
